# Children and adolescents with disorders of gut–brain interaction with comorbid hypermobility and orthostatic intolerance have worse outcomes

**DOI:** 10.1002/jpn3.70417

**Published:** 2026-03-19

**Authors:** Neha Santucci, Austin VonAxelson, Li Jesse, Graham Kahleb, Jennifer Hardy, Megan Miller, Rashmi Sahay

**Affiliations:** 1 Pediatric Gastroenterology, Cincinnati Children’s Hospital Medical Center, Cincinnati, Ohio, USA; 2 Department of Pediatrics, University of Cincinnati College of Medicine, Cincinnati, Ohio, USA; 3 Internal Medicine, University of Cincinnati College of Medicine, Cincinnati, Ohio, USA; 4 Behavioral Medicine and Clinical Psychology, Cincinnati Children’s Hospital Medical Center, Cincinnati, Ohio, USA; 5 Biostatistics and Epidemiology, Cincinnati Children’s Hospital Medical Center, Cincinnati, Ohio, USA

**Keywords:** functional, gastrointestinal, hypermobile Ehlers–Danlos syndrome, postural orthostatic tachycardia syndrome

## Abstract

**Objectives:**

Disorders of gut–brain interaction (DGBI) affect about 40% of children and are often comorbid with hypermobility spectrum disorders (HSDs) and orthostatic intolerance (OI). However, how these comorbidities impact outcomes in pediatric DGBI is not well understood. This study aimed to compare outcomes in DGBI patients with HSD, OI, both, or neither.

**Methods:**

We reviewed records of patients aged 9–21 years from a multidisciplinary DGBI clinic. Patients met Rome IV criteria for DGBI and had documented HSD and/or OI diagnoses from specialists in gastroenterology, rheumatology, genetics, cardiology, adolescent medicine, and others. HSD terms included Ehlers–Danlos syndrome, hypermobile Ehlers–Danlos syndrome, and generalized hypermobility; OI terms included postural orthostatic tachycardia syndrome, dysautonomia, and orthostatic hypotension. Clinical data included the following validated questionnaires: abdominal pain index, nausea severity scale, functional disability inventory, patient health questionnaire-9 (Depression), children somatization inventory, pediatric insomnia severity index, pain catastrophizing scale for children, and screen for child anxiety related disorders (Anxiety). We compared DGBI patients with both HSD and OI, those with either disorder, and those without.

**Results:**

Of 175 patients, 46% had HSD and 43% had OI. Patients with both HSD and OI had significantly worse nausea, depression, disability, and somatization scores than others (*p* < 0.01). HSD and OI groups individually also showed worse outcomes than non-HSD/non-OI groups. Moderate correlations were found between depression and anxiety in OI and nausea and disability in HSD.

**Conclusions:**

Comorbid HSD and OI worsen DGBI symptoms. Accurate diagnosis and treatment are critical to improving outcomes due to shared autonomic dysfunction.

## 1 | INTRODUCTION

Disorders of gut–brain interaction (DGBI) comprise a wide array of abdominal pain predominant irritable bowel syndrome, functional dyspepsia, abdominal migraine, and functional abdominal pain not otherwise specified, and nonpain predominant disorders (cyclic vomiting syndrome, functional constipation, and functional nausea/vomiting). The exact prevalence of DGBI in the pediatric population is difficult to define. In a cross-sectional study of children aged 0–18 using Rome IV criteria, 25% of children and adolescents met criteria for DGBI.^1^ DGBI can be associated not only with pain, but can cause severe nausea, disability, sleep disturbances, anxiety, depression, and school absenteeism Interestingly, DGBI have been associated with other comorbid conditions like hypermobility spectrum disorder (HSD), Type III hypermobile Ehlers–Danlos syndrome (hEDS), and orthostatic intolerance (OI), including postural orthostatic tachycardia syndrome (POTS).^2^ HSD is a spectrum of connective tissue disorders with a broad severity and multiple system involvement, which includes joint hypermobility syndrome (JHS). However, Ehlers–Danlos syndrome (EDS) is a genetic connective tissue condition with more than 20 subtypes.^3^ hEDS is a specific subtype of EDS with multiple systemic symptoms with a hypermobility predominance.^3^ The 2017 consensus guidelines for EDS created the term HSD to identify patients who have some components of JHS that do not meet the stricter guidelines for hEDS. HSD can be further broken down into generalized, peripheral, localized, and historical HSD. Symptoms of HSD can include instability, chronic pain, joint laxity, mild stretchy skin, and even tissue manifestations. While hEDS and HSD are different conditions, they share many of the same symptoms and outcomes. HSD is diagnosed via the Beighton score plus one or more musculoskeletal findings. hEDS is diagnosed via Beighton scores and other Ehlers–Danlos criteria via exclusion of other conditions on the EDS spectrum.^3^ Outside of joint manifestations, some of the most debilitating symptoms of hEDS and HSD can include OI and DGBI.

OI is defined as sustained symptoms of lightheadedness, palpitations, fatigue, or hypotension experienced with postural changes. POTS is defined as sustained orthostatic tachycardia linked with dizziness, fainting, and often associated with other systems. OI is often associated with the same symptoms and multisystem involvement as POTS, but may not meet strict heart rate guidelines for formal diagnosis of POTS.^4^ Tilt testing is an often-used modality in diagnosis of OI. However, tilt testing in pediatrics has some limitations with a lack of standardization, time requirement, inaccurate results based on duration of testing, lack of broad-based availability, and insurance coverage. This suggests that symptoms may be a better diagnostic tool in these patients.

The association between HSD and OI is well established.^5^ However, little is understood about how DGBI outcomes differ in patients with HSD or OI. This is important since we often see overlap in the pediatric population, leading to severe disability. In fact, HSD and OI have challenging impacts on the pediatric population with increased rates of anxiety, depression, pain, and sleep difficulties. Children with hEDS or HSD have rates of anxiety and depression (up to 80% and 42%, respectively). This is associated with worse health-related quality of life.^6^ OI is also associated with anxiety, depression, and sleep disturbances, which can have an impact on social function and school performance (24). Given the shared characteristics and outcomes of these three conditions—HSD, OI, and DGBI, more information is needed to understand how outcomes differ concerning DGBI. Thus, we aimed to compare outcomes between pediatric DGBI patients with comorbid HSD or OI, as well as those with both conditions versus those without. We hypothesized that DGBI patients with comorbid HSD or OI would have worse outcomes and psychological functioning. As above, these comorbid conditions have significant impacts of pediatric depression, anxiety, sleep, pain, and somatization. Hence, we explored these outcomes for patients in a pediatric DGBI clinic.

## 2 | METHODS

### 2.1 | Ethics statement

This study strictly follows ethical guidelines as expected by the Cincinnati Children’s Hospital Institutional Review Board. This study received IRB approval (IRB# 2021–0844). Informed consent was provided before participants were included in the study and confidentiality was maintained through the study.

### 2.2 | Study design

Electronic medical records, including demographics and medical history of patients aged 9–21 years seen at the Cincinnati Children’s Hospital Medical Center multidisciplinary DGBI clinic, were reviewed from 2019 to 2022. Patients who met the Rome IV criteria for a DGBI with comorbid diagnosis of HSD including hEDS and/or OI were identified by reviewing broad medical documentation from specialists and multidisciplinary providers, including pediatric gastroenterology, rheumatology, genetics, cardiology, primary care, adolescent medicine, physical therapy, and nutrition. Search terms for HSD [M35.7] included EDS [Q79.6], hEDS [Q79.62], HSD, and generalized hypermobility. Search terms for OI [G90.A] were POTS [R63.32], orthostatic hypotension [I95.1], and non-cardiac syncope (in accordance with the American Academy of Pediatrics classification). Outcomes were compared using validated questionnaires collected as part of clinical care. The questionnaires used were:

Abdominal pain index^7^: Validated to evaluate the frequency, duration, and intensity of abdominal pain episodes in children, assessing pain severity and its impact on daily activities.

Nausea severity scale (NSS)^8^: Validated tool to measure the severity of nausea symptoms in children, including frequency, duration, and intensity.

Pain catastrophizing scale for children^9^: Validated to assess catastrophic thinking related to pain in children, measuring rumination, magnification, and helplessness associated with pain to understand the psychological impact of chronic pain.

Children’s somatization inventory (CSI)^10^: Validated to measure the extent of somatic symptoms in children and adolescents, evaluating the frequency of symptoms like stomachaches, headaches, and dizziness.

Functional disability inventory (FDI)^11^: Validated to measure perceived difficulty in performing daily activities due to physical health problems, assessing functional impairment in children with chronic pain conditions.

Pediatric insomnia severity index (PISI)^12^: Validated to assess the severity of insomnia symptoms in children, evaluating difficulty falling asleep, staying asleep, and overall sleep quality, to understand the impact of insomnia on daily functioning.

Screen for child anxiety related disorders (SCARED)^13^: Validated to screen for anxiety disorders in children and adolescents, including general anxiety, separation anxiety, social anxiety, and school phobia.

Patient health questionnaire-9 (PHQ-9)^14^: Validated to measure the severity of depressive symptoms in children and adolescents, including mood, interest in activities, sleep, and appetite, and is used for screening, diagnosis, and monitoring of depression.

### 2.3 | Statistical analysis

Categorical data were presented as frequency counts with group differences examined using Chi-square or Fisher’s Exact test. Continuous variables were summarized as mean (95% confidence interval) or median (interquartile range [IQR]). Group comparisons were done using either two-sample *t*-test or the Wilcoxon Rank Sum test.

## 3 | RESULTS

### 3.1 | Demographic data

Demographic details are presented in Table 1. Of 175 patients, 78 (45%) had HSD and 76 (43%) OI. 45 (26%) patients had both HSD and OI (Table 1). Mean ages in these groups were 15.9 years in the HSD group, 16.6 years in the OI group, and 16.6 years in the “both” HSD and OI group. In all three groups, the majority were females (91%, 90%, and 98%, respectively) and Caucasian (86%, 87%, and 89%, respectively). Gender and race differed between the groups.

### 3.2 | Diagnoses data

DGBI diagnoses details are presented in Supporting Information: Table S1. In the sampled cohort, the most common diagnoses in all groups.

### 3.3 | Outcomes between groups

**FIGURE 1:**
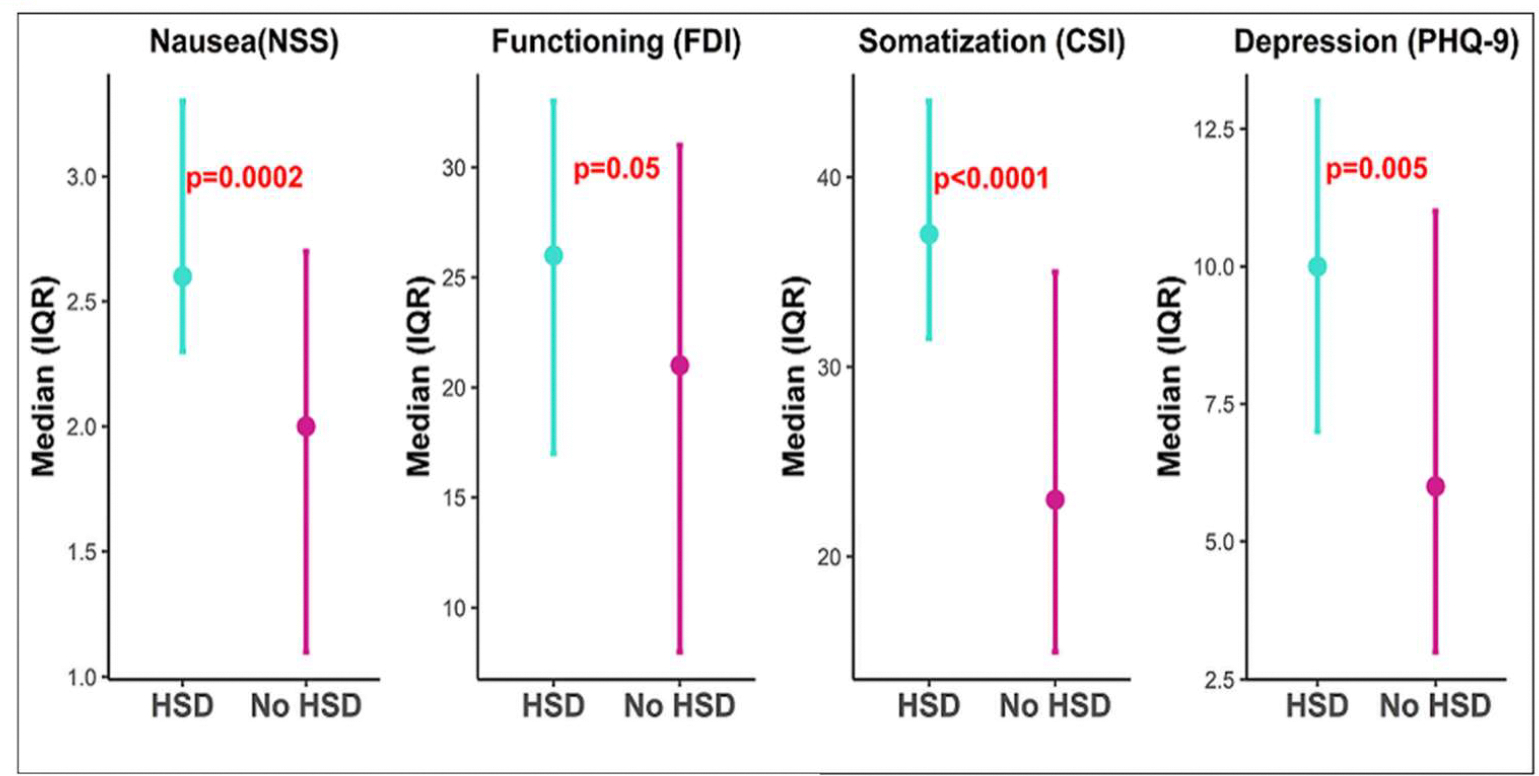
Outcomes HSD versus no HSD. Displays outcomes presented as median (IQR) comparing HSD versus non-HSD group. Groups compared by two-sample *t*-test and Wilcoxon Rank Sum Test. CSI, children’s somatization inventory; FDI, functional disability inventory; HSD, hypermobility spectrum disorder; IQR, interquartile range; NSS, nausea severity scale; PHQ-9, patient health questionnaire-9.

**FIGURE 2:**
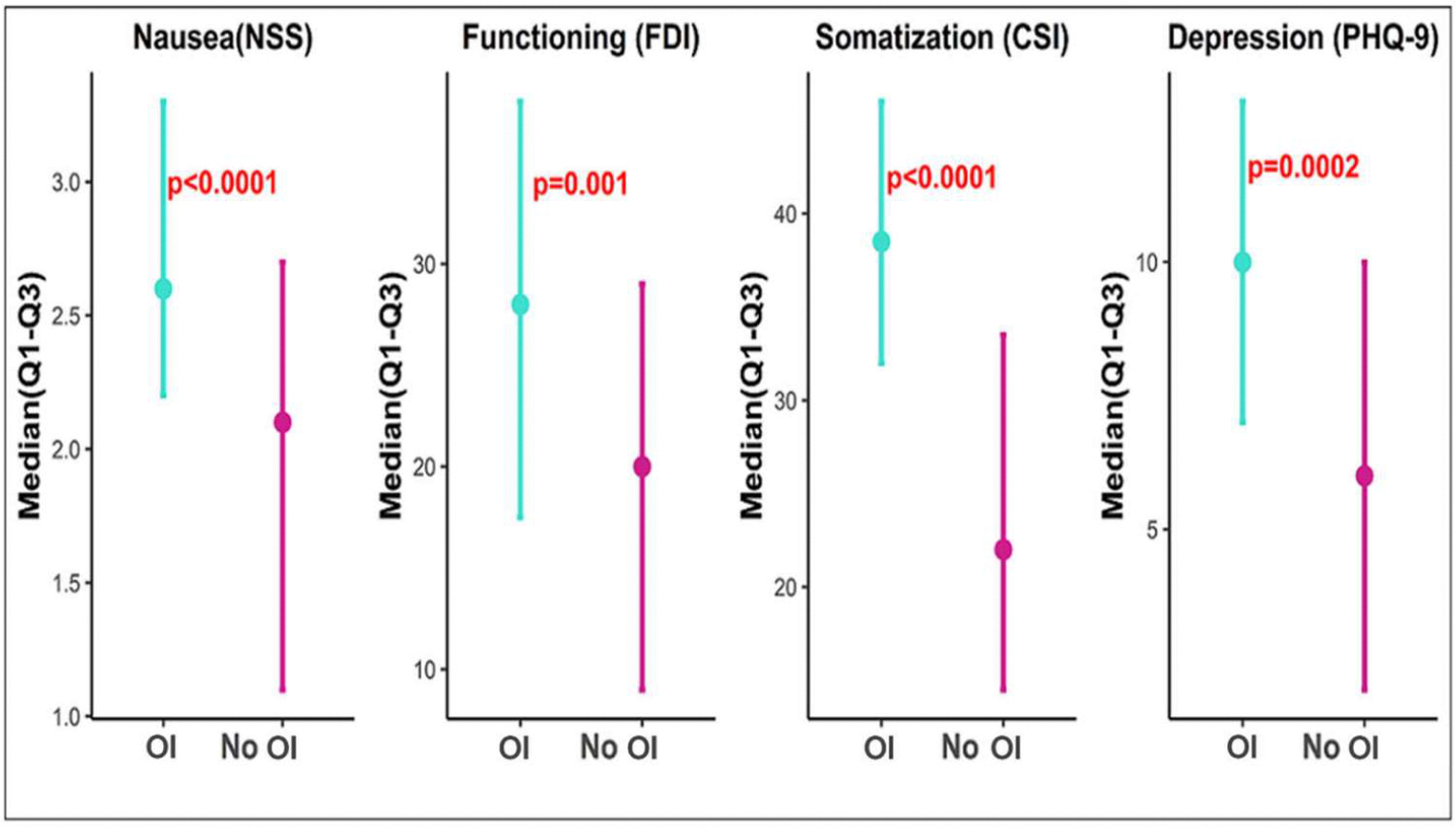
Outcomes OI versus no OI. Displays outcomes presented as median (IQR) comparing HSD versus non-HSD group. Groups compared by two-sample *t*-test and Wilcoxon Rank Sum Test. CSI, children’s somatization inventory; FDI, functional disability inventory; HSD, hypermobility spectrum disorder; IQR, interquartile range; NSS, nausea severity scale; OI, orthostatic intolerance; PHQ-9, patient health questionnaire-9.

**FIGURE 3:**
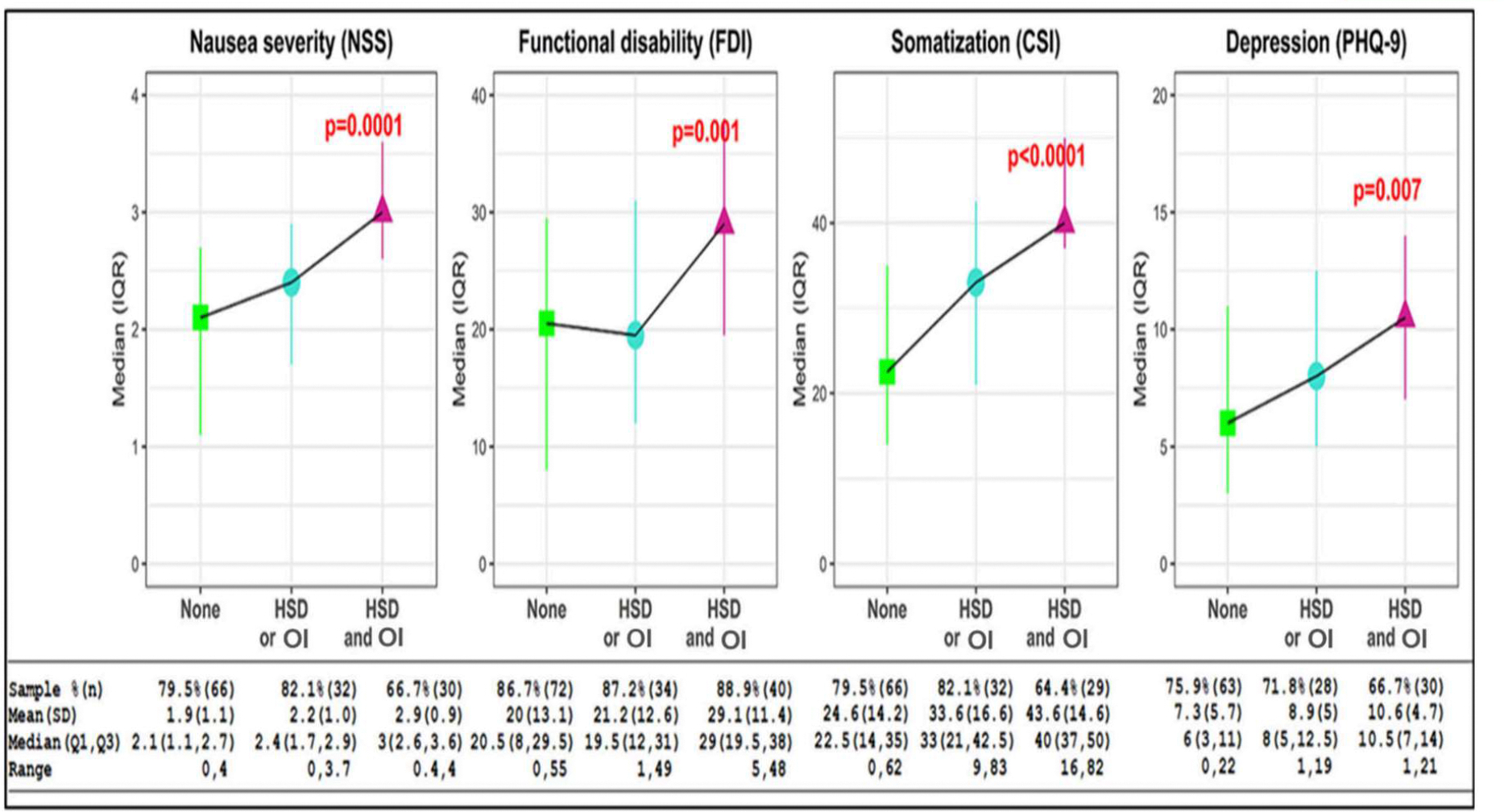
Outcomes three-way comparison of DGBI patients with both HSD/OI compared to either and none. Displays three HSD or OI, Either HSD or OI, or Both HSD and OI. Groups compared by Two-sample *t*-test and Wilcoxon Rank Sum Test. CSI, children’s somatization inventory; DGBI, disorders of gut-brain interaction; FDI, functional disability inventory; HSD, hypermobility spectrum disorder; IQR, interquartile range; NSS, nausea severity scale; OI, orthostatic intolerance; PHQ-9, patient health questionnaire-9.

Comparison of outcomes between the HSD versus without HSD (Figure 1), OI versus without OI (Figure 2), and those with both conditions is displayed in Figure 3. Patients with both HSD and OI had worse NSS (*p* < 0.0001), PHQ-9 (*p* = 0.007), FDI (*p* = 0.001), and CSI (*p* < 0.001) compared to patients with either OI or HSD and patients with neither (Figure 1). The same measures were worse in DGBI patients with HSD when compared to non-HSD as well as DGBI patients with OI compared to those without (*p* < 0.05, Table 1). The rest of the measures did not significantly differ between the groups (*p* > 0.05, Supporting Information: Table S2).

## 4 | DISCUSSION

This study compares the outcomes for patients with HSD and OI compared to those without in a DGBI clinic. We found that patients with HSD had worse nausea, somatization, depression, and disability compared to those without. Similarly, patients with OI had worse outcomes in the same metrics. Interestingly, we found that patients who had both conditions had more severe nausea, somatization, depression, and disability when compared to those with either of the conditions individually. Our study suggests a potential shared pathology linking HSD and OI, contributing to worse outcomes for patients with DGBI.

DGBIs are multifactorial with components of gut dysbiosis, visceral sensitivity, early-life events, and psychosocial factors.^15^ Symptoms such as nausea and abdominal pain worsen functioning and mental health symptoms, which further worsen gastrointestinal (GI) symptoms, setting up a vicious cycle that is hard to break.^16,17^ The mechanisms connecting HSD and OI are not well understood, but the association is clear. A study from 2020 utilized 2017 consensus criteria reported that 31% of OI patients in the cohort also met criteria for hEDS, with an additional 24% with HSD not meeting hEDS criteria.^18^

HSD, including hEDS, has multisystem involvement. Gastrointestinal symptoms are some of the most frequently experienced by patients with HSD. In fact, a recent study of 435 patients with hEDS/JHS reported a high prevalence of GI disorders like dysphagia (32%), constipation (61%), dyspepsia, and/or gastroparesis (25%).^19^ A large study from Priyadarshini et al. suggested there are multiple pathologies leading to GI symptoms for patients with HSD. Connective tissue abnormalities in HSD likely contribute to altered gut motility, gastroesophageal reflux, and autonomic dysfunction.^16^ Additionally, 52% of patients with HSD in this study were identified with comorbid gastroparesis, further connecting HSD to GI pathologies.

Autonomic dysfunction may explain the shared GI symptoms for OI and HSD patients. GI blood flow, motility, and secretion are regulated by an integral neural mechanism involving inputs from the central nervous system and the enteric nervous system. Abnormalities in the autonomic nervous system, primarily from vagal inefficiency, are suspected to cause excessive and uncoordinated gastrointestinal activity in patients with OI and HSD. Additionally, reduced vagal efficiency (perhaps related to abnormalities in connective tissue) also causes dysregulation of splanchnic circulation and capacity. This may explain worsened orthostatic symptoms in patients with OI after meals due to the increased pooling in the splanchnic vascular bed.^20^ A study on pediatric patients with chronic, unexplained nausea found increased prevalence of autonomic dysfunction. Moreover, studies targeting autonomic dysfunction in pediatric patients have found that treating underlying autonomic dysfunction in patients with OI leads to improvement in nausea and abdominal pain, further suggesting a connection between autonomic dysfunction and GI symptoms.^21^

Twenty-six percent of the patients in our cohort had both HSD and OI. Formal diagnosis of HSD and OI can be challenging and often requires involvement of multiple subspecialists like genetics, rheumatology, cardiology, gastroenterology, psychiatry, and so on. Studies comparing these groups run the risk of under-reporting diagnoses or including patients without the diagnosis. Strict clinical criteria are required using guidelines. More work is needed to better understand the mechanism connecting the GI symptoms of HSD and OI. For this study, we reviewed notes of subspecialists and ensured formal diagnoses by experts. If a patient had not been diagnosed with included diagnoses after review, they were excluded from these groups.

## 5 | CONCLUSION

In conclusion, patients with HSD and OI have worse nausea, depression, somatization, and disability than those without. Patients with both conditions have worse outcomes than those with the conditions independently. This suggests a common pathophysiology like autonomic dysfunction or vagal insufficiency. An advantage of this study is that it is one of the early studies comparing the GI outcomes of patients with HSD and OI. This study further strengthens the hypothesis of a shared pathology between these conditions and their impact on the GI tract. A limitation of this study found that there is often a lack of screening and diagnosis of these conditions, as they are often diagnoses of exclusion, and underdiagnosed in this population. It requires a multidisciplinary team to identify cases of HSD and OI. Another limitation is that this is a retrospective study, and further analysis under a prospective model may provide additional insights.

Finally, we believe our findings suggest potential clinical applications for this population of patients. For children with DGBI, particularly with significant disability, providers should consider screening for symptoms of hypermobility and OI. When access to rheumatology, cardiology, and medical genetics are available, consider expert, multidisciplinary referrals. Treating symptoms of autonomic dysfunction can improve GI outcomes. It is important to manage other comorbidities when present, as these will improve outcomes overall, including GI symptoms.

## Supplementary Material

Supplemental Table 1

Supplemental Table 2

SUPPORTING INFORMATION

Additional supporting information can be found online in the Supporting Information section at the end of this article.

## Figures and Tables

**TABLE 1:** Demographic data.

Demographics	HSD (n = 78)	No HSD (17 = 91)	p-value	OI (n = 76)	No OI (77 = 99)	p-value	Both HSD and OI (77 = 45)	p-value

Age (years)	15.9 ± 2.3	16.2 ± 2.5	0.29	16.6 ± 1.8	15.7 ± 2.7	0.08	16.6 ± 1.5	0.04
Sex								
Male *n* (%)	7 (9.0)	36 (39.6)	<0.0001	8 (10.5)	36 (36.4)	<0.0001	1 (2–2)	<0.0001
Female *n* (%)	71 (91.0)	55 (60.4)		68 (89.5)	63 (63.6)		44 (97.8)	
Race								
White *n* (%)	67 (85.9)	80 (87.9)	0.76	66 (86.8)	86 (86.9)	0.29	40 (88.9)	0.13
Black *n* (%)	5 (6.4)	6 (6.6)		3 (4)	9 (9.1)		2 (4.4)	
Asian *n* (%)	3 (3.9)	1 (11)		3 (4)	1 (1)		3 (6.7)	
Unknown *n* (%)	3 (3.9)	4 (4.4)		4 (5.3)	3 (3)		0 (0)	

*Note:* Demographics represented in the study. Demographics were broken down into age, sex, and race. Categorical data were presented as frequency counts and percentages, and group differences examined using Chi-Square or Fisher's Exact test. Bold values indicate statistical significance.

Abbreviations: HSD, hypermobility spectrum disorder; OI, orthostatic intolerance.

## What is Known

- Hypermobility spectrum disorder and orthostatic Intolerance are associated with poor health outcomes in the pediatric population.
- Hypermobility and orthostatic intolerance are often associated with disorders of gut–brain interaction (DGBI).

### What is New

- Pediatric patients with DGBI and either hypermobility or orthostatic intolerance had worse nausea, depression, and disability than those without.
- Pediatric patients with DGBI and both hypermobility and orthostatic intolerance had even worse nausea, depression, and disability than those with one alone.
