# Supplementary material for: Children and adolescents with disorders of gut–brain interaction with comorbid hypermobility and orthostatic intolerance have worse outcomes
Source: J Pediatr Gastroenterol Nutr. Author manuscript; Available in PMC 2026 Jul 8. (PMC13238288; doi:10.1002/jpn3.70417)
Supplement: Supplemental Table 1 [file NIHMS2167355-supplement-Supplemental_Table_1.docx]

**Supplemental Table 1: DGBI Diagnoses**

| **DGBI Diagnosis** | **HSD**  **(n=78)** | **No HSD**  **(n=91)** | **P-value** | **OI**  **(n=76)** | **No OI**  **(n=99)** | **P-value** | **Both HSD and OI**  **(n=45)** | **P-Value** |
| --- | --- | --- | --- | --- | --- | --- | --- | --- |
| Functional Dyspepsia [n(%)] | 50 (64.1) | 39 (42.9) | **0.0058** | 52 (68.4) | 40 (40.4) | **0.00020** | 31 (68.9) | **0.0221** |
| IBS [n(%)] | 55 (70.5) | 49 (53.9) | **0.026** | 51 (67.1) | 59 (59.6) | 0.31 | 30 (66.7) | **0.021** |
| Rumination [n(%)] | 6 (7.7) | 7 (7.7) | 1.00 | 7 (9.2) | 6 (6.1) | 0.43 | 1 (2.2) | **0.0309** |
| Abdominal Migraine [n(%)] | 0 (0) | 3 (3.3) | 0.25 | 1 (1.3) | 2 (2) | 1.00 | 0 (0) | 0.434 |
| CVS [n(%)] | 4 (5.1) | 6 (6.6) | 0.75 | 4 (5.3) | 6 (6.1) | 1.00 | 3 (6.7) | 1 |
| Functional Constipation [n(%)] | 1 (1.3) | 3 (3.3) | 0.63 | 3 (4) | 1 (1) | 0.32 | 1 (2.2) | 0.8089 |
| Functional N/V [n(%)] | 1 (1.3) | 4 (4.4) | 0.38 | 1 (1.3) | 3 (3) | 0.63 | 1 (2.2) | 0.5122 |
| Functional Abdominal Pain (NOS) [n(%)] | 12 (15.4) | 16 (17.6) | 0.70 | 10 (13.2) | 20 (20.2) | 0.22 | 8 (17.8) | 0.7522 |
| Other [n(%)] | 5 (6.4) | 2 (2.2) | 0.25 | 5 (6.6) | 3 (3) | 0.30 | 3 (6.7) | 0.4639 |

*HSD: Hypermobility Spectrum Disorder, OI: Orthostatic Intolerance, IBS: Irritable Bowel Syndrome, CVS: Cyclic Vomiting Syndrome, Functional N/V: Functional Nausea/Vomiting, Functional Abdominal Pain (NOS): Functional Abdominal Pain Not Otherwise Specified
