# Supplementary material for: Children and adolescents with disorders of gut–brain interaction with comorbid hypermobility and orthostatic intolerance have worse outcomes
Source: J Pediatr Gastroenterol Nutr. Author manuscript; Available in PMC 2026 Jul 8. (PMC13238288; doi:10.1002/jpn3.70417)
Supplement: Supplemental Table 2 [file NIHMS2167355-supplement-Supplemental_Table_2.docx]

**Supplemental Table 2: Outcomes of Patients with HSD vs without HSD, with OI vs. without OI, and with HSD and OI**

| **Questionnaire**  **[Median(IQR)]** | **HSD**  **(n=78)** | **No HSD**  **(n=91)** | **P-value** | **OI**  **(n=76)** | **No O**  **(n=99)** | **P-value** | **Both HSD and OI**  **(n=45)** | **P-Value** |
| --- | --- | --- | --- | --- | --- | --- | --- | --- |
| Nausea (NSS) | 2.6 (2.3, 3.3) | 2.0 (1.1, 2.7) | **0.002** | 2.6(2.2, 3.3) | 2.1 (1.1, 2.7) | **<.0001** | 3 (2.6, 3.6)) | **.0001** |
| Functioning (FDI) | 55 (70.5) | 49 (53.9) | **0.026** | 51 (67.1) | 59 (59.6) | 0.31 | 29 (19.5, 38) | **0.0012** |
| Somatization (CSI) | 6 (7.7) | 7 (7.7) | 1.00 | 7 (9.2) | 6 (6.1) | 0.43 | 40 (37, 50) | **<.0001** |
| Depression (PHQ-9) | 0 (0) | 3 (3.3) | 0.25 | 1 (1.3) | 2 (2) | 1.00 | 10.5 (7, 14) | **0.007** |
| Pain Catastrophizing (PCS-C) | 21 (15, 27) | 21 (11, 30) | 0.97 | 23 (15, 29) | 19.5 (9, 27) | 0.1 | 25 (18,30) | 0.11 |
| Insomnia (PISI) | 14 (9.5, 20.5) | 13  (7, 20) | 0.23 | 14 (9, 21) | 12 (8, 17.5) | 0.085 | 15 (13, 21) | 0.24 |
| Anxiety (SCARED) | 27.5 (16, 42) | 21 (9, 40) | 0.24 | 28.5 (15.5, 44) | 20.5 (9, 37) | 0.09 | 29 (14-44) | 0.53 |
| Abdominal Pain (API) | 2.9 (2, 3.4) | 2.7 (1.7, 3.5) | 0.58 | 2.5 (1.9, 3.5) | 2.8  (1.8, 3.5) | 0.77 | 3 (2, 3.6) | 0.37 |

*HSD: Hypermobility Spectrum Disorder, O: Postural Orthostatic Tachycardia Syndrome, NSS: Nausea Severity Scale, FDI: Functional Disability Index, CSI: Children’s Somatization Inventory, PHQ-9: Patient Health Questionnaire-9, PCS-C: Pain Catastrophizing Scale for Children, PISI: Pediatric Insomnia Severity Index, SCARED: Screen for Child Anxiety Related Disorders, API: Abdominal Pain Index
